# Editorial: Career development in the educational system

**DOI:** 10.3389/fpsyg.2023.1205957

**Published:** 2023-05-24

**Authors:** Anna Parola, Jenny Marcionetti, Francesco Sulla, Lawrence P. W. Wong

**Affiliations:** ^1^Department of Humanities, University of Naples Federico II, Naples, Italy; ^2^Department of Education and Learning, University of Applied Science and Arts of Southern Switzerland, Locarno, Switzerland; ^3^Department of Humanities, University of Foggia, Foggia, Italy; ^4^Department of Counseling and Psychology, Hong Kong Shue Yan University, Hong Kong, China

**Keywords:** career transitions, career choices, adolescents, career development, teacher career-related behavior

## 1. Introduction

Making career choices represents the most demanding developmental task in adolescence and early adulthood. Today's society, mainly characterized by the absence of a predetermined career path and uncertainty and flexibility in the labor market, makes it increasingly difficult for adolescents and young people looking for future careers.

The educational system, as a social elevator responsible for the formative and human growth of individuals, may be an active promoter of career development. As a holding environment, schools have the critical function of “scaffolding” student career transitions. Educational systems could help to strengthen and promote career skills, competence, and resources, as well as adolescents' awareness of the choices available and foster students' optimism and hope about their future (Fantinelli et al., 2023; Parola et al., 2023). Moreover, individuals might be more active in the creation of their careers, in the direction of their active lives, and in choosing the type of role that they have in their society (Fusco et al., 2021).

This Research Topic gathers different contributions from different parts of the world on career development in the educational system. In the studies examined, attention was mainly paid to certain aspects of career development, such as career exploration (Demulder et al.), career expectations (Cadilhe et al.), and career engagement (Petruziello et al.). In a strong manner, the importance of interventions and career guidance in the educational setting has been stressed as beneficial for students to better cope with the challenges of the decision-making process. Finally, Western Countries Vocational Education and Training (VET) policies were also addressed, identifying the best practices in VET as building blocks (Romero-Rodriguez et al.) for the construction of an integrated career guidance plan.

In the Eastern side of the world, this collection of empirical studies showcased how modern career theories (Zhang et al.) and assessment tools (Song et al.; Yang et al.) were being locally adapted to facilitate the school-to-work transition of students with diverse abilities and school levels. This is critical to the overall future positive development of the student population because, like many places around the world, the career planning of students in the East has been significantly disrupted by the COVID-19 pandemic (Wong, 2022).

It is hoped that insights drawn from this research collection will be able to serve as a valuable reference for teachers and policy makers to reflect on how the provision of career-related teacher support in schools could be enhanced (Wong et al., 2022).

In conclusion, we thank all the authors and reviewers who contributed to the realization of this Research Topic and we hope that the reader will find it a useful reference for the state-of-the-art in career development in the education system.

## Author contributions

All authors listed have made a substantial, direct, and intellectual contribution to the work and approved it for publication.

## References

[B1] Fantinelli S. Esposito C. Carlucci L. Limone P. Sulla F. (2023). The influence of individual and contextual factors on the vocational choices of adolescents and their impact on wellbeing. Behav. Sci. 13, 233. 10.3390/bs13030233 36975258 PMC10045217

[B2] Fusco L. Parola A. Sica L. S. (2021). Life design for youth as a creativity-based intervention for transforming a challenging World. Front. Psychol. 12:662072. 10.3389/fpsyg.2021.662072 34017294 PMC8129198

[B3] Parola A. Pettignano M. Marcionetti J. (2023). Development and validation of the teacher career-related support self-efficacy (TCSSE) questionnaire. Behav. Sci. 13, 36. 10.3390/bs13010036 36661608 PMC9854607

[B4] Wong L. P. W. (2022). Issues concerning the interpretation and assessment of career adaptability: perspectives from Hong Kong, China. Youth. 2, 181–194. 10.3390/youth2020014

[B5] Wong L. P. W. Yuen M. T. Chen G. (2022). Career guidance and counselling: The nature and types of career-related teacher social support in Hong Kong secondary schools. Br. J. Guid. Counsel. 50, 897–915. 10.1080/03069885.2022.2040005

